# Case report: Double L611S/V617L *JAK2* mutation in a patient with polycythemia vera originally diagnosed with essential thrombocythemia

**DOI:** 10.3389/fonc.2022.937362

**Published:** 2022-11-22

**Authors:** Xiao-qing Li, Jia-jun Liu, Cheng-cheng Liu

**Affiliations:** ^1^ Department of Hematology, The Third Affiliated Hospital of Sun Yat-Sen University, Guangzhou, Guangdong, China; ^2^ Sun Yat-Sen Institute of Hematology, Guangzhou, Guangdong, China

**Keywords:** polycythemia vera, essential thrombocythemia, myeloproliferative neoplasm, *JAK2* L611S/V617L mutation, *CSF1R* mutation, phenotype transformation

## Abstract

Double *JAK2* mutations have rarely been described in myeloproliferative neoplasms (MPNs) and are demonstrated to be associated with the polycythemia vera (PV) phenotype. Here, we first report a case of a PV patient with a *de novo* double L611S/V617L *in cis* mutation of *JAK2*. A 40-year-old woman was admitted to the hospital with massive splenomegaly, multiple splenic infarcts, and abdominal pain. She had a 4-year history of erythrocytosis with an antecedent 10-year history of thrombocytosis before coming to our hospital. She was diagnosed with *JAK2* L611S/V617L double-mutant PV after a detailed medical examination in 2017. According to the literature, IFNα therapy can induce clinical, hematological, histopathological, and occasionally molecular remission in individuals with MPNs. Our report demonstrates that combination therapy with ruxolitinib and IFNα can lead to a substantial reduction in *JAK2* L611S/V617L double-mutant allele burden.

## Introduction

Somatic *JAK2* mutations are the most common disease-causing events in patients with myeloproliferative neoplasms (MPNs). Double *JAK2* mutations have rarely been described. A *JAK2* L611V missense mutation that presented *in cis* with V617F has been reported in three adult polycythemia vera (PV) patients, which resulted in the *JAK2* L611V/V617F double mutation (1). The coexistence of *JAK2* L611S and *JAK2* V617F variants was described in one adult PV patient who carried the *JAK2* L611S mutation *in cis* with the *JAK2* V617F mutation (2). A double L611S/V617F *JAK2* mutation was detected in a pediatric patient with PV (3). These double *JAK2* mutations were associated with a PV phenotype. Acquisition of additional mutations in hematopoietic stem cells (HSCs), which creates either compound-mutant *JAK2* alleles or a compound-heterozygous state (4), could influence the subclones and affect the disease phenotype in MPNs.

Here, we first report a case of a PV patient with a *de novo JAK2* L611S/V617L double mutation *in cis*. A 4-year history of erythrocytosis with an antecedent 10-year history of thrombocytosis in this case suggests that essential thrombocythemia (ET) and PV are a continuum of one basic disease. Sørensen et al. had reported promising 2-year end-of-study results of the clinical trial investigating combination treatment with ruxolitinib and low-dose pegylated interferon-α2 (PEG-IFNα2), with improved cell counts, reduced bone marrow cellularity and fibrosis, decreased *JAK2* V617F burden, and reduced symptom burden with acceptable toxicity in several patients with PV or myelofibrosis (5). In our case, the combination of ruxolitinib and IFNα could improve treatment efficacy and produced a good outcome.

## Case description

In January 2003, a woman suffered her first seizure. The initial hemogram showed a high platelet count (517 × 10^9^/L) and a normal hemoglobin concentration, hematocrit level, and white blood cell count. She had no special medical, family, or psychosocial history. A diagnosis of epilepsy was made, and carbamazepine was prescribed by the doctor at the local hospital as seizure treatment. While the patient was taking carbamazepine for epilepsy from January 2003 to May 2009, her hemogram showed a moderately high platelet count (maximum value: 659 × 10^9^/L). However, she did not have another blood test until November 2010.

In November 2010, her hemogram test showed a much higher platelet count (798 × 10^9^/L) and a slightly elevated hemoglobin concentration (157 g/L), hematocrit level (47.20%), and white blood cell count (11.66×10^9^/L). The bone marrow characteristics are the increase and clustering of pleiomorphic megakaryocytes with multi-lobulated nuclei and proliferation of myelopoiesis and erythropoiesis in a marked hypercellular bone marrow. Abdominal ultrasound examination revealed a mildly enlarged spleen (thickness, 4.4 cm; length, 11.8 cm). *BCR-ABL1* fluorescence in situ hybridization (FISH) showed a negative result. The patient was clinically diagnosed with ET without comprehensive genetic analysis for MPNs at the local hospital after exclusion of secondary causes for ET and received intermittent hydroxyurea therapy because of poor treatment co*MPL*iance. During the intermittent hydroxyurea treatment, the patient’s hemogram showed fluctuating, moderately high platelet counts (370–593 × 10^9^/L).

Due to poor treatment co*MPL*iance, the patient did not receive hydroxyurea therapy from September 2011 to October 2017. A single hemogram test in October 2014 showed a high hemoglobin concentration (172 g/L), hematocrit level (55.5%), platelet count (393 × 10^9^/L), and white blood cell count (10.7 × 10^9^/L).

In November 2017, the patient required emergency medical treatment for abdominal pain. The hemogram showed a high hemoglobin concentration (182 g/L), hematocrit level (57%), platelet count (347 × 10^9^/L), and white blood cell count (12.97 × 10^9^/L). Abdominal MRI revealed giant splenomegaly (thickness, 8.6 cm; length, 23.5 cm), multifocal hemosiderin deposition, and multiple infarcts in the spleen. The patient had a 4-year history of erythrocytosis with an antecedent 10-year history of thrombocytosis before coming to our hospital. Her medical, family, or psychosocial histories were otherwise unremarkable. As such, she was directly transferred from the outpatient clinic to the inpatient department for further medical examination.

**Figure 1:**
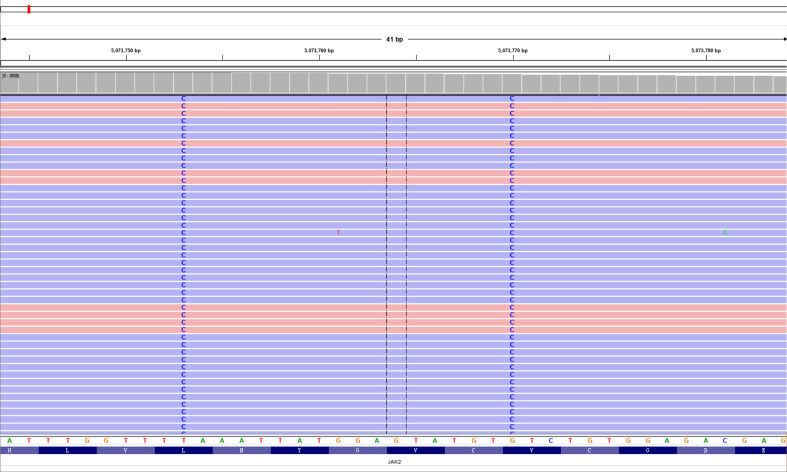
Double L611S/V617L *JAK2* mutation. Visualization of the two single-nucleotide variants in the aligned sequencing data using the Integrative Genomics Viewer (IGV) tool.

After detailed hematologic examination in November 2017, the patient’s bone marrow morphological examination revealed hyperplasia of all three lineages with megakaryocyte hyperplasia with micromegakaryocytes. Reticular fiber staining showed mild increase in reticular fibers (grade 0 myelofibrosis). The bone marrow sa*MPL*es were investigated by flow cytometry. Flow cytometric analysis of HLA-DR, CD33, CD34, CD117, CD10, CD56, CD19, CD5, CD7, CD2, CD11b, CD15, CD13, CD16, CD71, CD41, and CD45 revealed no obvious expression disorder. There was no detection of abnormal blasts and the immunophenotypic characteristics analyzed by flow cytometry were normal. Chromosomal analyses of the bone marrow showed a normal karyotype. *BCR-ABL1* fusion gene variants (p190, p210, p230, and rare variants) were not detected by reverse transcription-polymerase chain reaction (RT-PCR). Rearrangements of the *PDGFRA, PDGFRB, or FGFR1* genes were not detected by FISH analysis. There was no detection of a *JAK2* V617F mutation, *JAK2* exon 12 mutation, *MPL* W515L mutation, *MPL* W515K mutation, or a *CALR*eticulin (*CALR*) exon 9 deletion or insertion by PCR with Sanger sequencing. We then performed next-generation sequencing (NGS) for whole exome sequencing (WES) of her bone marrow cells. This revealed *de novo* double mutations in *JAK2*, including the *JAK2* V617L mutation (c.1849G>C; p.V617L), with a variant allele frequency (VAF) of 44.0%, and the *JAK2* L611S mutation (c.1832T>C; p.L611S), with a VAF of 44.7%. She also had a colony-stimulating factor 1 receptor (*CSF1R*) mutation (c.1460C>T; p.A487V; VAF 46.2%). Neither the double L611S/V617L *JAK2* mutation nor other MPN-associated mutations were detected in her buccal mucosal cells by NGS-WES, but the *CSF1R* A487V mutation was detected with a VAF of 46.2%, and thus proven to be germline. The Integrative Genomics Viewer visualization tool showed that both *JAK2* variants were located in the same reads (**Figure 1**). Cranial MRI revealed ischemic degeneration in the bilateral frontal cortex. Gene mutations associated with epilepsy were not detected.

The patient was diagnosed with *JAK2* L611S/V617L double-mutant PV after a detailed medical examination, and she received continuous treatment with ruxolitinib from November 2017 and combination therapy with ruxolitinib and IFNα from January 2021 (**Table 1**). During the follow-up time from November 2017, the hemoglobin concentration, hematocrit level, platelet count, and white blood cell count returned to normal 10 weeks after treatment initiation. Significant reductions of 45% and 56% in the cross-sectional area of the spleen were achieved 12 weeks (spleen: thickness, 6.3 cm; length, 17.5 cm) and 2 years later (spleen: thickness, 5.7 cm; length, 15.7 cm), respectively. Alleviation of the constitutional symptoms including excessive and disabling fatigue, early satiety, anorexia, night sweats, abdominal pain and discomfort, and cognitive co*MPL*aints had improved her quality of life 6 months later. VAFs of both the *JAK2* L611S and *JAK2* V617L mutations were 44%, 15%, and 11% in November 2017, November 2019, and November 2020, respectively, which suggested that these mutations had occurred in the same clone. The addition of IFNα to ruxolitinib therapy substantially decreased *JAK2* L611S/V617L allele burden. After the patient received the combined treatment of IFNα and ruxolitinib for 6 months, the VAF of the double L611S/V617L *JAK2* mutation was reduced from 11% to 5% (**Table 2**). During the follow-up period from November 2017 to November 2020 (ruxolitinib alone), ruxolitinib improved the patient’s splenomegaly and other symptoms, including early satiety, bloating, portal hypertension, fatigue, and undesired weight loss, which improved her overall quality of life.

**Table 1:** Clinical and biological characteristics of this patient with the double L611S/V617L *JAK2* mutation.

Time	January 2003	January 2003 to May 2009	November 2010	November 2010 to September 2011	October 2011 to October 2017	November 2017	December 2017 to December 2020	January 2021~
Diagnosis	Epilepsy	Epilepsy	ET Epilepsy	ET Epilepsy	ET switching to PV in 2014?Epilepsy	PV Epilepsy	PV Epilepsy	PV Epilepsy
PLT (×10^9^/L)	517	659	798	370–593	October 2014: 393	347	Normal	Normal
HGB (g/L)	142	Normal	157	Normal	October 2014: 172	182	Normal	Normal
HCT	40.0%	Normal	47.2%	Normal	October 2014: 55.5%	57.0%	Normal	Normal
Treatment	CBZ	CBZ	HU CBZ	HU CBZ	CBZ	Ruxolitinib CBZ	Ruxolitinib CBZ	IFNα + Ruxolitinib CBZ

WBC, white blood cell; HGB, hemoglobin; HCT, hematocrit; PLT, platelet; ET, essential thrombocythemia; PV, polycythemia vera; HU, hydroxyurea. CBZ, carbamazepine.

**Table 2:** Next-generation sequencing screening result of this patient.

Gene	Mutation	Exon	VAF (2017)	VAF (2019)	VAF (2020)	VAF (2021)
*JAK2*	NM_004972.3:c.1849G>C(p.V617L)	exon14	44.0%	15.2%	11.0%	5.6%
*JAK2*	NM_004972.3:c.1832T>C(p.L611S)	exon14	44.7%	15.5%	11.4%	5.4%
*CSF1R*	NM_005211.3:c.1460C>T(p.A487V)	exon10	46.2%	47.0%	50.1%	49.5%

VAF, variant allele frequency.

## Diagnostic assessment

According to the new World Health Organization (WHO) 2016 criteria (6), the patient was clinically diagnosed with ET in November 2010. However, the ET may have existed in January 2003 considering disease evolution and medical history. The data in November 2017 confirmed a diagnosis of PV with a double *JAK2* L611S/V617L mutation according to the WHO 2016 criteria for PV. Massive splenomegaly and multiple splenic infarcts are poor prognostic factors.

## Discussion

To our knowledge, this is the first report of a double *JAK2* L611S/V617L mutation in PV. The *JAK2* L611S mutation alone was detected in a child with a germline mutation associated with thrombocytosis (7). The presence of the *JAK2* L611S mutation leads to the constitutive activation of *JAK2*/STAT signaling, even in the absence of erythropoietin receptor expression (8). The *JAK2* V617L mutation was detected in an individual with ET, and germline fibroblast testing confirmed the somatic origin of the mutation (9). *JAK2* V617L and *JAK2* V617I mutations have previously been shown to induce cytokine independence and constitutive downstream signaling in Ba/F3 cells randomly mutated at position 617 of *JAK2* (10).

The double *JAK2* L611V/V617F mutation increases the activation of *JAK2*, AKT, and ERK1/2 (but not of STAT5) and is associated with isolated erythrocytosis (1). We speculate that a similar mechanism could underlie the effects of double *JAK2* L611S/V617L mutation.

The studies by Van Egeren et al. (9) and Williams et al. (11) showed that the latency between the acquisition of the driver gene mutation and the manifestation of MPN was much longer than that was generally assumed. This provides fascinating insights into the early steps in the pathogenesis of MPN, which might raise opportunities for earlier intervention and provide a new paradigm for cancer development.

Studies have revealed that the *JAK2* V617F allele burden, differential signal transducer and activator of transcription activation, order of other somatic mutations, and interindividual genetic variation influence the predisposition to specific MPN subtypes (12). However, the specific role of the double *JAK2* L611S/V617L mutation in our PV patient with a history of ET is unclear.

The presence of different mutations within the same gene often results in different biological properties that make it challenging for us to deeply understand the *de novo* double *JAK2* L611S/V617L *in cis* mutation in PV. This patient experienced a decrease in the *JAK2* L611S/V617L double mutation allele burden under the combination therapy of ruxolitinib and IFNα. The *de novo JAK2* L611S/V617L double mutation *in cis* identified in this case might be an oncogenic mutation or driver mutation, as *JAK2* V617F was discovered in 2005 as the driver mutation of the majority of non-*BCR-ABL1* MPNs (13, 14).

The patient in this study had a germline *CSF1R* mutation (c.1460C>T; p.A487V). Unlike the reported pathogenic germline *CSF1R* mutations located within the PTK domain (15), the mutation identified in our patient was located within the immunoglobulin domain. The clinical significance of the *CSF1R* A487V mutation remains unknown. The dominant transmembrane receptor controlling the differentiation and survival of almost all macrophages regardless of their origins is the colony-stimulating factor 1 receptor. Although the *CSF1R* A487V germline mutation is known to be nonpathogenic, we speculate that the presence of *JAK2* mutations in HSCs and endothelial cells, bone marrow-derived macrophages, or yolk sac-derived brain-specific macrophages with the *CSF1R* A487V mutation might cooperatively promote multiple splenic infarcts and ischemic degeneration in the bilateral frontal cortex (probably microthrombosis), which likely triggered her seizures. These seizures might be TIAs provoked by the increased number of more adhesive thrombocytes.

HSC heterogeneity underlies the disparate phenotypes of MPNs harboring the same initiating mutation, and malignant transformation of neoplasms might involve a specific subset of stem cells within a heterogeneous stem cell population. In ET, HSCs with *JAK2* mutations are more sensitive to IFN signaling to megakaryocyte differentiation and show strong megakaryocyte lineage priming (9). Furthermore, hematopoietic stem and progenitor cells (HSPCs) with *JAK2* mutations show a lineage bias towards the megakaryocyte-erythroid fate and contribute to the development of thrombosis (12). IFNα therapy can induce clinical, hematological, histopathological, and occasionally molecular remission in individuals with MPNs. Some results suggested that upon treatment (including long course therapeutic dose of IFNα), the mutant megakaryocyte-primed HSC population was reduced by promoting apoptosis or quiescence of the mutant cells (12). Clinical evaluation of pegylated IFNα-2a and ruxolitinib in a phase II study of PV and MF showed substantial reductions of *JAK2* V617F allele burden with 41% of patients showing a molecular response along with improved cytosis and fibrosis as well as acceptable toxicity (5). These findings demonstrate that pegylated IFNα adds substantial clone suppression to ruxolitinib therapy and that the combination with ruxolitinib improves tolerability of IFNα therapy. These results suggested that our patient would benefit from sustained combination therapy with ruxolitinib and IFNα.

A recent study confirmed that therapeutic targeting of Y-Box Binding Protein 1 (YBX1)-dependent ERK signaling in combination with *JAK2* inhibition could eradicate cells harboring mutations in *JAK2* (16). The ruxolitinib/nilotinib/prednisone combination showed synergistic inhibitory effects on the JAK/STAT and MAPK signaling pathways in MPN cells (17, 18). Thus, IFNα combined with *JAK2* or MEK inhibition might improve therapeutic efficacy.

Given the lifelong trajectories of MPNs in humans and considering the underlying mechanisms in MPNs, the heterogeneity of *JAK2* mutant HSCs and HSPCs, and clonal expansion and evolution, studies to increase the understanding of MPNs and to improve our management of MPN patients are needed.

## Data availability statement

The original contributions presented in the study are included in the article/supplementary materials. Further inquiries can be directed to the corresponding author.

## Ethics statement

The studies involving human participants were reviewed and approved by The Third Affiliated Hospital of Sun Yat-Sen University. The patients/participants provided their written informed consent to participate in this study.

## Author contributions

X-QL participated in the clinical care, analyzed the data, and was a major contributor in writing the manuscript. J-JL conducted the literature search and revised the manuscript. C-CL conducted the study, provided patient care, analyzed the data, and contributed with final manuscript drafting. All authors contributed to the article and approved the submitted version.

## Funding

The project is funded by GuangZhou Basic and Applied Basic Research Foundation, the grant number is 202201010973.

## Acknowledgments

We thank all the investigators, including the physicians, nurses, pathologists, and laboratory technicians in this study.

## Conflict of interest

The authors declare that the research was conducted in the absence of any commercial or financial relationships that could be construed as a potential conflict of interest.

## Publisher’s note

All claims expressed in this article are solely those of the authors and do not necessarily represent those of their affiliated organizations, or those of the publisher, the editors and the reviewers. Any product that may be evaluated in this article, or claim that may be made by its manufacturer, is not guaranteed or endorsed by the publisher.
